# Efficacy and Safety of Empagliflozin Continuation in Patients with Type 2 Diabetes Hospitalised for Acute Decompensated Heart Failure

**DOI:** 10.3390/jcm10163540

**Published:** 2021-08-12

**Authors:** Luis M. Pérez-Belmonte, Michele Ricci, Jaime Sanz-Cánovas, Mercedes Millán-Gómez, Julio Osuna-Sánchez, M. Isabel Ruiz-Moreno, M. Rosa Bernal-López, María D. López-Carmona, Manuel Jiménez-Navarro, Juan J. Gómez-Doblas, José P. Lara, Ricardo Gómez-Huelgas

**Affiliations:** 1 Servicio de Medicina Interna, Hospital Regional Universitario de Málaga, Instituto de Investigación Biomédica de Málaga (IBIMA), Universidad de Málaga (UMA), 29010 Málaga, Spain; michele.ricci4@gmail.com (M.R.); jaimesc25@hotmail.com (J.S.-C.); mruiz.salud@gmail.com (M.I.R.-M.); mdlcorreo@gmail.com (M.D.L.-C.); ricardogomezhuelgas@hotmail.com (R.G.-H.); 2 Unidad de Neurofisiología Cognitiva, Centro de Investigaciones Médico Sanitarias (CIMES), Facultad de Medicina, Universidad de Málaga (UMA), 29010 Málaga, Spain; julioosunas@gmail.com (J.O.-S.); jplara@uma.es (J.P.L.); 3 Servicio de Medicina Interna, Hospital Helicópteros Sanitarios, 29660 Marbella, Spain; mechimillan@hotmail.com; 4 Centro de Investigación Biomédica en Red Enfermedades Cardiovasculares (CIBERCV), Instituto de Salud Carlos III, 28029 Madrid, Spain; mjimeneznavarro@gmail.com (M.J.-N.); jjgomezdoblas@gmail.com (J.J.G.-D.); 5 Servicio de Medicina Interna, Hospital Comarcal de La Axarquía, 29700 Vélez-Málaga, Spain; 6 Centro de Investigación Biomédica en Red Fisiopatología de la Obesidad y Nutrición (CIBERobn), Instituto de Salud Carlos III, 28029 Madrid, Spain; 7 Unidad de Gestión Clínica Área del Corazón, Hospital Universitario Virgen de la Victoria, Instituto de Investigación Biomédica de Málaga (IBIMA), Universidad de Málaga (UMA), 29010 Málaga, Spain

**Keywords:** type 2 diabetes, heart failure, empagliflozin, hospitalization

## Abstract

There is little evidence on the use of sodium−glucose cotransporter 2 inhibitors in hospitalised patients. This work aims to analyse the glycaemic and clinical efficacy and safety of empagliflozin continuation in patients with type 2 diabetes hospitalised for acute decompensated heart failure. This real-world observational study includes patients treated using our in-hospital antihyperglycaemic regimens (basal-bolus insulin vs. empagliflozin-basal insulin) between 2017 and 2020. A propensity matching analysis was used to match a patient on one regimen with a patient on the other regimen. Our primary endpoints were the differences in glycaemic control, as measured via mean daily blood glucose levels, and differences in the visual analogue scale dyspnoea score, NT-proBNP levels, diuretic response, and cumulative urine output. Safety endpoints were also analysed. After a propensity matching analysis, 91 patients were included in each group. There were no differences in mean blood glucose levels (152.1 ± 17.8 vs. 155.2 ± 19.7 mg/dL, *p* = 0.289). At discharge, NT-proBNP levels were lower and cumulative urine output greater in the empagliflozin group versus the basal-bolus insulin group (1652 ± 501 vs. 2101 ± 522 pg/mL, *p* = 0.032 and 16,100 ± 1510 vs. 13,900 ± 1220 mL, *p* = 0.037, respectively). Patients who continued empagliflozin had a lower total number of hypoglycaemic episodes (36 vs. 64, *p* < 0.001). No differences were observed in adverse events, length of hospital stay, or in-hospital deaths. For patients with acute heart failure, an in-hospital antihyperglycaemic regimen that includes continuation of empagliflozin achieved effective glycaemic control, lower NT-proBNP, and greater urine output. It was also safer, as it reduced hypoglycaemic episodes without increasing other safety endpoints.

## 1. Introduction

Heart failure (HF) is one of the leading causes of hospital admissions around the world and entails a high risk of early postdischarge mortality and rehospitalisation [1,2].

In recent years, multiple randomised clinical trials have reported that sodium−glucose cotransporter 2 (SGLT2) inhibitors significantly reduce hospitalisations for acute decompensated HF in patients with type 2 diabetes (T2D) [3,4,5,6,7]. Recently, the SGLT2 inhibitor dapagliflozin has been shown to lead to reductions in the risk of death and hospitalisations due to HF in patients with HF with a reduced ejection fraction regardless of the presence of T2D [8]. These benefits may be partly explained by its effect on diuresis/natriuresis and its favourable effects on the cardiometabolic and renal systems [9].

In non-critically ill patients with T2D, a multidose insulin regimen that involves once-daily basal insulin and bolus of rapid-acting insulin before meals [10,11] and, most recently, the use of dipeptidyl peptidase-4 (DPP-4) inhibitors alone or in combination with basal insulin [12,13,14,15,16,17,18,19] are the preferred therapies for treating in-hospital hyperglycaemia. However, to date, little evidence exists on the use of SGLT2 inhibitors in hospitalised patients. Recently, two prospective randomised controlled studies evaluated the effects of empagliflozin on clinical outcomes in patients with acute decompensated HF [20,21]. Empagliflozin was found to be safe and improved HF outcomes in hospitalised patients. Recently, results have been reported from one clinical trial which evaluated the efficacy and safety of sotagliflozin in patients with chronic HF with a recent episode of HF decompensation (SOLOIST) [22]. Its primary composite endpoint of the total number of deaths due to cardiovascular causes and hospitalizations and urgent visits due to HF showed a reduction of 33% in patients treated with sotagliflozin compared to a placebo. Currently, there are several ongoing, randomized placebo-control trials in patients with and without T2D after admission for HF, evaluating the clinical benefit and safety of in-hospital use of SGLT2 inhibitors: empagliflozin (EMPULSE) [23], and dapagliflozin (DAPA ACT HF-TIMI 68 [24] and Dapagliflozin Heart Failure Readmission [25]). Based on the beneficial therapeutic profile of SGLT2 inhibitors and the experience of their use in non-hospitalised patients, we aimed to retrospectively analyse the glycaemic and clinical efficacy and safety of empagliflozin continuation in combination with basal insulin compared to a basal-bolus insulin regimen in patients with T2D hospitalised for acute decompensated HF. Our hypothesis was that the empagliflozin regimen would have positive effects on glycaemic control and clinical outcomes and would be safe in patients with T2D patients hospitalised for acute decompensated HF.

## 2. Material and Methods

### 2.1. Study Design and Patients

We conducted a real-world observational study on patients with T2D admitted for acute decompensated HF to 4 hospitals in Málaga, Spain (Hospital Regional Universitario de Málaga, Hospital Universitario Virgen de la Victoria, Hospital Helicopteros Sanitarios, and Hospital Cenyt) from January 2017 to December 2020. Investigators from each hospital reviewed each patient’s electronic medical records in order to collect patient data.

Acute HF was defined as all of the following: dyspnoea at rest or on minimal exertion, signs of congestion (peripheral oedema, rales, and/or congestion on chest X-ray), N-terminal pro-brain natriuretic peptide (NT-proBNP) ≥1400 pg/mL (≥2000 pg/mL in patients with atrial fibrillation), and intravenous treatment with loop diuretics.

Non-critically ill patients with T2D hospitalised for acute decompensated HF and treated with empagliflozin for at least 3 months prior to the hospitalisation were selected. According to our in-hospital antihyperglycaemic protocol, patients can be treated with two possible regimens: a basal-bolus insulin regimen or an empagliflozin-basal insulin regimen. The basal-bolus insulin regimen is the conventional glucose-lowering treatment used in the hospital setting and is recommended for all patients as a standard of care regardless of their admission blood glucose level. This regimen includes the discontinuation of empagliflozin and the initiation of once-daily basal insulin (insulin glargine (Lantus; Sanofi-Aventis, Gentilly, France), administered at 04:00 p.m.) and rapid-acting insulin analogues before meals (insulin lispro (Humalog; Eli-Lilly, Indianapolis, IN, USA) or insulin aspart (Novorapid; Novo Nordisk, Bagsvaerd, Denmark). Alternatively, patients with an admission blood glucose (BG) level of less than 250 mg/dL who do not meet any exclusion criteria have the option to continue with empagliflozin (Jardiance; Boehringer Ingelheim, Ingelheim am Rhein, Germany). This regimen involves a single dose at the same dose as prior to hospitalisation (10 or 25 mg) administered at 09:00 a.m. in addition to a once-daily basal insulin (insulin glargine (Lantus; Sanofi-Aventis, Gentilly, France), administered at 04:00 p.m.). The exclusion criteria for empagliflozin use are: signs of ketoacidosis and/or hyperosmolar hyperglycaemic state, type 1 diabetes, concomitant hospital treatment with a systemic glucocorticoid, expected admission to an intensive care unit, planned cardiac surgery, acute renal function impairment with an estimated glomerular filtration rate (based on the Chronic Kidney Disease Epidemiology Collaboration formula ) [26] ≥ 45 mL/min/1.73 m^2^, clinically-relevant liver disease or cirrhosis, blood dyscrasias or any disorders causing haemolysis or unstable red blood cells, gastrointestinal obstruction, those expected to be without oral intake, use of artificial nutrition (enteral or parenteral), urinary tract infection, genital infection, perineal necrotizing fasciitis, acute peripheral vascular disease, and pregnant or nursing (lactating) women. The choice of which regimen to use is made by physicians according to their clinical judgment.

The insulin dose is calculated according to admission BG levels, age, and serum creatinine and is modified during hospitalisation when required (basal or fasting hyperglycaemia or hypoglycaemia). When supplemental rapid-acting insulin is required before meals and bedtime, the dose is calculated according to BG levels, total daily insulin units, and the patient’s weight. Fasting, preprandial, and bedtime capillary BG levels are measured using a point-of-care glucose meter. Additionally, BG levels are measured any time a patient experiences symptoms of hypoglycaemia or when requested by the healthcare provider. Level 1 hypoglycaemia is defined as a measurable BG level <70 mg/dL and ≥54 mg/dL, level 2 hypoglycaemia as a measurable BG level <54 mg/dL, and level 3 hypoglycaemia as a severe event characterised by an altered mental and/or physical status requiring assistance, according to the American Diabetes Association criteria [27]. When patients on the empagliflozin regimen experience treatment failure, they are switched to the basal-bolus insulin regimen. Treatment failure is defined as either two consecutive measurements or a mean daily BG level >250 mg/dL. The target of therapy is to maintain fasting and preprandial glucose levels within a range of 140–180 mg/dL.

### 2.2. Study Outcomes

Our primary endpoint was to analyse the differences in glycaemic control, as determined by mean daily BG levels; BG levels at mealtime and bedtime; BG levels 100–140 mg/dL, 140–180 mg/dL, and 180–250 mg/dL; number and day of treatment failures; total daily insulin dose (basal and prandial); and number of daily insulin injections between the empagliflozin-basal insulin and basal-bolus insulin regimens in patients with T2D admitted for acute decompensated HF. In addition, we analysed the differences between regimens in regard to the visual analogue scale (VAS), dyspnoea score and the NT-proBNP levels from baseline (at admission) to discharge; diuretic response, defined as weight loss (kilograms) per 40 mg furosemide or equivalent at discharge; and cumulative urine output during hospitalisation.

Safety endpoints included total adverse events (general hospital complications including those of special interest, such as liver damage, worsening renal function, ketoacidosis and/or hyperosmolar hyperglycaemic state), worsening HF (defined as worsening signs and/or symptoms of HF that require intensification of intravenous therapy for HF or mechanical ventilation, renal support, or circulatory support), adverse events that lead to the discontinuation of empagliflozin (excluding the discontinuation of empagliflozin due to treatment failure), hypoglycaemic episodes (total number, number of patients with 1 or ≥2 episodes, hypoglycaemia incidence rates and number of patients with hypoglycaemia (levels 1, 2 and 3), length of hospital stay and in-hospital deaths.

### 2.3. Statistical Analysis

Statistical analyses were performed using SAS for Windows, version 9.3 (SAS, Cary, NC, USA), and SPSS Statistics for Windows, version 15.0 (SPSS, Chicago, IL, USA).

In order to match each patient who started on the basal-bolus insulin regimen with a patient who started on the empagliflozin-basal insulin regimen in a 1:1 manner, a propensity score with a caliper of 0.2 and a greedy matching algorithm were used. The probability of starting the empagliflozin-basal insulin regimen (as opposed to the basal-bolus insulin regimen) was estimated using a logistic regression model that included variables that could have affected treatment assignment or outcomes as independent variables (sex; smoking and alcohol use disorder status; history of hypertension; dyslipidaemia; chronic kidney disease; cerebrovascular disease; chronic obstructive pulmonary disease; liver disease; atrial fibrillation; coronary artery disease; VAS dyspnoea score at admission, New York Heart Association (NYHA) functional classification; left ventricular ejection fraction; principal cause of heart failure; prior hospitalization for heart failure, heart failure medication; amount of time the patient has had T2D; BG, serum creatinine, estimated glomerular filtration rate, NT-proBNP, and transaminase levels at admission; body mass index; and at-home treatment). In order to assess the adequacy of propensity matching, we used the standardised difference (SD) of patient characteristics after matching. A significant imbalance in the group was considered to be present if an SD between baseline variables of higher than 10% was found.

Baseline characteristics were analysed using descriptive statistics. Continuous and categorical variables were shown as means ± standard deviation and as absolute value and percentage, respectively. The hypoglycaemia incidence rate per 100 patient-years was calculated. The differences between groups were determined using the two-sample Student’s t-test or the Mann-Whitney U test for continuous variables and Pearson’s χ2 test for categorical variables. Values were considered to be statistically significant when *p* < 0.05. Multiple comparisons across different days of therapy were adjusted conservatively using Tukey’s adjustment.

### 2.4. Ethics Approval and Consent to Participate 

Only patients who had previously given consent for their medical records to be used for medical research were included. Data confidentiality and patient anonymity were maintained at all times, in accordance with Spanish regulations on observational studies. Patient identifying information was deleted before the database was analysed; it is not possible to identify patients on an individual level either in this article or in the database. This study was carried out in accordance with the Declaration of Helsinki and was approved by the Institutional Research Ethics Committee of Málaga on 27 October 2016 (Ethics Committee code: REDIME 27-10-2016).

## 3. Results

**Figure 1:**
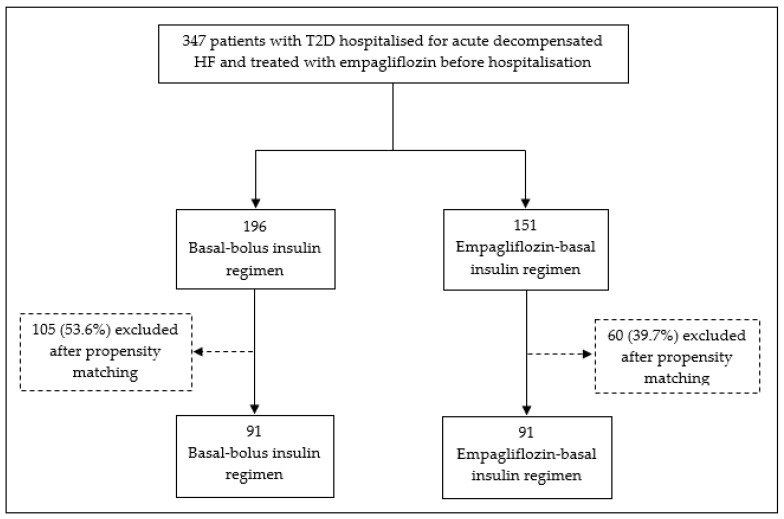
Patient flow charts for basal-bolus insulin regimen versus empagliflozin-basal insulin regimen. T2D: Type-2 diabetes.

A total of 347 patients with T2D hospitalised for acute decompensated HF and treated with empagliflozin before hospitalisation were included. Of them, 196 (56.5%) discontinued empagliflozin and started the basal-bolus insulin regimen and 151 (43.5%) continued empagliflozin at the same dose as prior to hospitalisation (65 and 86 patients at doses of 10 mg and 25 mg, respectively) in combination with basal insulin. After propensity matching, 91 patients were included in each group. In the empagliflozin group, 40 patients had a 10-mg dose and 51 patients had a 25-mg dose before hospitalisation. A flow chart for patient inclusion for both regimens is shown in Figure 1.

The baseline clinical characteristics of patients were well balanced between groups following the propensity matching analysis, with standardised differences of <10%. Before the propensity matching analysis, patients who continued the empagliflozin were younger and had a higher NYHA functional class. These data are shown in Table 1.

In regard to the glycaemic control, there were no differences between the basal-bolus insulin and the empagliflozin-basal insulin regimen groups in mean daily BG levels during hospitalisation; BG levels at mealtime and bedtime; BG levels 100–140 mg/dL, 140–180 mg/dL, and 180–250 mg/dL; and the number and day of treatment failures after the propensity matching analysis. However, the total insulin dose and number of injections per day during hospitalisation were significantly lower in the empagliflozin-basal insulin group compared to the basal-bolus insulin group. Total basal and supplemental rapid-acting insulin doses did not differ significantly between the treatment groups. Before matching, patients managed with the empagliflozin-basal insulin regimen had a higher mean BG level during hospitalisation, mean BG level before lunch, a mean BG 180–250 mg/dL, and number of treatment failures than those managed with the basal-bolus insulin regimen. Similar to the data found on the post-propensity matching analysis, patients managed with the basal-bolus insulin regimen received more total insulin and a greater number of injections per day. No differences were observed in total basal and supplemental rapid-acting insulin doses. These data are summarised in Table 2.

**Figure 2:**
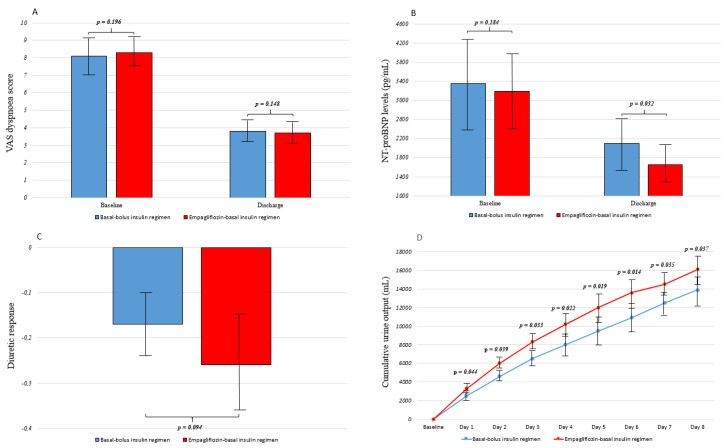
Visual analogue scale dyspnoea score (**A**), NT-proBNP levels (**B**), diuretic response (**C**), and cumulative urine output (**D**) according to the antihyperglycaemic regimen. Differences between regimens in regard to visual analogue scale dyspnoea score (**A**) and NT-proBNP levels (**B**) from baseline (at admission) to discharge, diuretic response (**C**) (defined as body weight loss (kilograms) per 40 mg furosemide or equivalent) at discharge, and cumulative urine output (**D**) during hospitalisation are shown. Variables are shown as means ± standard deviation. Values were considered to be statistically significant when *p* < 0.05. NT-proBNP: N-terminal pro-brain natriuretic peptide; VAS: visual analogue scale.

The VAS dyspnoea score and NT-proBNP levels declined during hospitalisation. At discharge, there was no difference in the VAS dyspnoea score between groups (3.8 ± 0.7 vs. 3.7 ± 0.6, *p* = 0.148), but NT-proBNP levels were lower in the empagliflozin-basal insulin group than in the basal-bolus insulin group (1652 ± 501 vs. 2101 ± 522 pg/mL, *p* = 0.032). Moreover, although no significant difference was found in the diuretic response between groups (−0.17 ± −0.07 vs. −0.26 ± −0.10, *p* = 0.094), the cumulative urine output was significantly greater in patients treated with empagliflozin compared with basal-bolus insulin during the hospitalisation (at discharge: 16,100 ± 1510 vs. 13,900 ± 1220 mL, *p* = 0.037). These results are shown in Figure 2. The mean loop diuretic dose (shown as the equivalent dose of furosemide) through discharge was 140 ± 60 mg furosemide in the basal-bolus insulin regimen group and 120 ± 60 mg in the empagliflozin-basal insulin regimen group (*p* = 0.348). No significant differences were observed in blood pressure levels between groups.

In regard to safety endpoints, there were no differences in adverse events (total, adverse events of special interest, or worsening HF), length of hospital stay (range: 4–12 days; 95.5% of patients between 5 and 8 days), or in-hospital deaths between groups after the propensity matching analysis. Six patients (6.6%) had adverse events that led to the discontinuation of empagliflozin. The empagliflozin-basal insulin group had a lower total number of hypoglycaemic episodes, patients with 1 or ≥2 episodes, hypoglycaemia incidence rate, and patients with any level 1 hypoglycaemia. Before matching, patients on the basal-bolus insulin regimen had more cardiovascular events and instances of worsening HF, though the total number of adverse events did not differ between the groups. All hypoglycaemic episodes analysed were significantly more frequent in patients on the basal-bolus insulin regimen than those on the empagliflozin-basal insulin regimen. The length of hospital stay and number of in-hospital deaths were similar between the treatment groups. Safety endpoints are summarised in Table 3.

## 4. Discussion

This real-world study found that in patients with T2D hospitalised for acute decompensated HF, the in-hospital continuation of empagliflozin in combination with basal insulin was as efficacious in regards to glycaemic control as a conventional multidose basal-bolus insulin regimen. In addition, the empagliflozin regimen increased the cumulative urine output and reduced the NT-proBNP levels during hospitalisation but led to no differences in the VAS dyspnoea score or diuretic response between groups. Finally, the empagliflozin-basal insulin regimen was found to be safer than the conventional basal-bolus insulin regimen, as there were fewer hypoglycaemic episodes. No differences were observed in adverse events, length of hospital stay, or in-hospital deaths.

To our knowledge, this work is the first real-world study addressing the safety and glycaemic and clinical efficacy of empagliflozin continuation during the hospitalisation of patients with acute decompensated HF. To date, two randomised controlled clinical trials with small sample sizes have evaluated the effects of empagliflozin on clinical outcomes in patients hospitalised for acute decompensated HF [20,21]. The first study (EMPA-RESPONSE-AHF Study), which was a placebo-controlled trial on 80 patients with and without T2D conducted by Damman et al. [20], reported that empagliflozin increased urine output and reduced a composite endpoint of worsening HF, rehospitalisation for HF, or death at 60 days from admission. No benefits in regards to dyspnoea (assessed by the VAS dyspnoea score), diuretic response, NT-proBNP, or length of hospital stay were found. More recently, Tamaki et al. [21] reported a second clinical trial that evaluated the effect of empagliflozin as an add-on therapy in 59 patients with T2D hospitalised for acute decompensated HF. They found that compared to patients on conventional glucose-lowering therapy, those treated with empagliflozin had effective decongestion, as well as a significant reduction in percent change in plasma output between baseline and later time points. In addition, serum NT-proBNP was lower and hemoconcentration was more frequent in the empagliflozin group than in the conventional treatment group. These data are in accordance with our findings. We found that empagliflozin significantly increased urine output from the beginning of hospitalisation. Patients on empagliflozin-basal insulin had a mean cumulative urine output that was 2200 mL higher at day eight than patients in whom empagliflozin was discontinued. We also found that NT-proBNP levels were lower in the empagliflozin-basal insulin group than in the basal-bolus insulin group at discharge. No differences were found in the VAS dyspnoea score or diuretic response. The disparity between these indices of decongestion, coupled with the fact that there was no effect on signs and symptoms of volume overload, may be related to the limited correlation observed between these variables in clinical practice [28].

Unlike chronic HF, there are currently no therapies available that improve the prognosis of acute decompensated HF. The use of loop diuretics to reduce congestion remains the mainstay of acute HF treatment [28]. Several drugs have been evaluated, but no benefits on clinical outcomes have been reported. Moreover, most of them have shown significant adverse effects on blood pressure and renal function [29,30,31]. However, based on the current evidence, empagliflozin has been shown to increase the diuretic effect without increasing adverse events [20,21].

Both randomised clinical trials on this subject were focused on clinical endpoints and no data on glycaemic control were reported [20,21], despite the fact that hyperglycaemia has been associated with adverse outcomes among hospitalised patients [32]. Basal-bolus insulin and dipeptidyl peptidase-4 (DPP-4) inhibitors alone or in combination with insulin have been shown to lead to significant improvements in glycaemic control and reductions in complications in hospitalised patients with T2D [9,10,11,12,13,14,15,16,17,18,19]. In our study, in addition to clinical endpoints, we analysed differences in glycaemic control and found that in-hospital continuation of empagliflozin in combination with basal insulin was as efficacious as a multidose basal-bolus insulin regimen in regard to glycaemic control. The empagliflozin-basal insulin regimen was also simpler, with a lower total daily insulin dose and fewer daily injections. Several studies comparing DPP-4 inhibitor regimens to basal-bolus insulin regimens have shown similar glycaemic benefits and these regimens have simplified the in-hospital management of T2D [11,12,13,14,15,16,17,18,19].

Regarding safety endpoints, both the Damman et al. [20] and Tamaki et al. [21] studies showed that empagliflozin was safe, as there was no increase in adverse events or deaths during hospitalisation. In our study, the use of empagliflozin was also safe in the hospital setting. Moreover, we observed a significant reduction in hypoglycaemic episodes. Insulin regimens have been linked to a higher risk of hypoglycaemic episodes, which occur in as many as one out of every three treated patients [33]. Previous studies on the in-hospital use of DPP-4 inhibitors also observed this reduction in hypoglycaemic episodes, providing important data on in-hospital safety [14,15,17,18,19].

SGLT2 inhibitors have shown clear benefits in HF hospitalisations in patients with [3,4,5,6,7] and without T2D [8]. However, their benefits in patients with acute decompensated HF have yet to be established. In this study, we found that empagliflozin may be appropriate and safe to continue in patients with decompensated HF in the hospital setting.

Although our findings are important, this study has several limitations. First, given the retrospective nature of our data, the use of a propensity matching analysis cannot rule out the possibility of unmeasured confounding factors. Second, continuation of empagliflozin in patients who are hospitalised is not fully implemented in our area. In fact, most patients were managed with the conventional basal-bolus insulin regimen and discontinued empagliflozin. Third, there was no standardised protocol for the treatment of acute decompensated HF and no protocol for diuretic therapy, which could have influenced our findings. Fourth, due to the relatively low number of events and complications, their relationship to the antihyperglycaemic regimen could not be conclusively determined. Fifth, only empagliflozin continuation was evaluated in this study. The reasons behind this choice are that a significant percentage of patients are treated with empagliflozin in our area and we wanted to evaluate its efficacy and safety in isolation, without the interference of other SGLT2 inhibitors. An interesting future line of research would be an evaluation of continuing other SGLT2 inhibitors during hospitalisation. Lastly, we only included patients admitted for acute decompensated HF, but the efficacy and safety of empagliflozin could be assessed in other groups of patients hospitalised for other cardiovascular diseases. Further research is required in order to confirm these results and provide more evidence. 

## 5. Conclusions

We found that an in-hospital antihyperglycaemic regimen that includes the continuation of empagliflozin achieved effective glycaemic control and was associated with greater cumulative urine output and lower NT-proBNP levels than a conventional basal-bolus insulin regimen in patients with T2D hospitalised for acute decompensated HF. In addition, the empagliflozin regimen was found to be safer, as there were fewer hypoglycaemic episodes and no differences in adverse events, length of hospital stay, or in-hospital deaths. Large, randomised trials with SGLT2 inhibitors are required to provide more evidence on the efficacy and safety of SGLT2 inhibitors in patients with acute decompensated HF.

## Figures and Tables

**Table 1:** Clinical characteristics of patients hospitalised for acute decompensated heart failure according to antihyperglycaemic regimen: pre- and post-propensity matching analysis.

	Pre-Propensity Matching Analysis				Post-Propensity Matching Analysis			
	Basal-Bolus (*n* = 196)	Empagliflozin-Basal (*n* = 151)	Standardised Difference	*p*-Value	Basal-Bolus (*n =* 91)	Empagliflozin-Basal (*n* = 91)	Standardised Difference	*p*-Value
Age (years)	73.6 ± 6.2	70.2 ± 5.4	0.127	0.034	72.7 ± 5.8	72.0 ± 5.6	0.009	0.349
Women	106 (54.1%)	71 (47.0%)	0.109	0.044	48 (52.7%)	46 (50.5%)	0.011	0.401
Body weight (kg)	88.9 ± 8.3	90.9 ± 11.7	0.042	0.154	89.4 ± 8.6	90.0 ± 10.9	0.003	0.451
Body Mass Index (kg/m^2^)	28.9 ± 1.6	29.8 ± 2.5	0.020	0.198	29.0 ± 1.8	29.4 ± 2.4	0.008	0.417
Body Mass Index ≥30	64 (32.7%)	51 (33.8%)	0.037	0.277	30 (33.0%)	30 (33.0%)	0.001	0.554
Abdominal circumference (cm)	94.5 ± 7.0	98.0 ± 10.0	0.071	0.103	96.0 ± 7.4	96.9 ± 8.9	0.006	0.389
Systolic blood pressure (mmHg)	136.5 ± 14.3	130.4 ± 13.5	0.047	0.152	134.1 ± 14.0	132.9 ± 13.8	0.022	0.178
Diastolic blood pressure (mmHg)	72.6 ± 8.4	68.9 ± 8.0	0.036	0.144	70.8 ± 8.2	69.1 ± 8.1	0.010	0.270
Diabetes duration (years)	9.2 ± 3.4	8.5 ± 3.3	0.074	0.102	9.0 ± 3.4	8.8 ± 3.3	0.009	0.425
Diabetes therapy at admission								
Monotherapy	28 (14.3%)	20 (13.2%)	0.059	0.121	13 (14.3%)	12 (13.2%)	0.014	0.268
Combination of oral glucose—lowering drugs	168 (85.7%)	131 (86.8%)	0.059	0.121	78 (85.7%)	79 (86.8%)	0.015	0.249
Biguanide	123 (62.8%)	92 (60.9%)	0.022	0.201	57 (62.6%)	56 (61.5%)	0.008	0.344
Sulfonylurea	20 (10.2%)	16 (10.6%)	0.020	0.211	9 (10.0%)	9 (10.0%)	0.002	0.559
Dipeptidyl peptidase-4 inhibitor	49 (25.0%)	36 (23.8%)	0.024	0.226	22 (24.2%)	22 (24.2%)	0.002	0.472
Glucagon-like peptide-1 receptor agonist	59 (30.1%)	50 (33.1%)	0.041	0.147	28 (30.8%)	30 (33.0%)	0.017	0.222
Insulin therapy	51 (26.0%)	36 (23.8%)	0.048	0.155	23 (25.3%)	22 (24.2%)	0.012	0.274
Chronic Heart failure	141 (71.9%)	113 (74.8%)	0.039	0.134	67 (73.6%)	68 (74.7%)	0.015	0.257
NYHA functional classification			0.109	0.044			0.024	0.194
II	137 (69.9%)	96 (63.6%)			61 (67.0%)	59 (64.8%)		
III	54 (27.5%)	53 (35.1%)			28 (30.8%)	30 (33.0%)		
IV	5 (2.6%)	2 (1.3%)			2 (2.2%)	2 (2.2%)		
Left ventricular ejection fraction	47.5 ± 23.0	45.7 ± 24.8	0.048	0.157	47.0 ± 23.0	46.8 ± 24.1	0.009	0.377
Left ventricular ejection fraction <40%	164 (42.8%)	111 (43.7%)	0.011	0.252	63 (43.2%)	63 (43.2%)	0.001	0.519
Principal cause of heart failure			0.035	0.114			0.026	0.185
Ischemic	99 (50.5%)	78 (51.7%)			46 (50.5%)	47 (51.6%)		
Nonischemic	79 (40.3%)	60 (39.7%)			37 (40.7%)	36 (39.6%)		
Unknown	18 (9.2%)	13 (8.6%)			8 (8.8%)	8 (8.8%)		
Prior hospitalization for heart failure	101 (51.5%)	84 (55.6%)	0.051	0.131	48 (52.7%)	50 (54.9%)	0.020	0.227
Heart failure medication								
Loop diuretic	173 (90.0%)	131 (86.8%)	0.039	0.137	81 (89.0%)	79 (86.8%)	0.029	0.201
Thiazide diuretic	20 (10.2%)	17 (11.2%)	0.018	0.233	10 (11.0%)	10 (11.0%)	0.011	0.298
Other diuretic	4 (2.0%)	3 (2.0%)	0.029	0.190	-	-	-	-
Angiotensin-converting enzyme inhibitor	88 (45.0%)	65 (43.0%)	0.030	0.177	40 (44.0%)	40 (44.0%)	0.004	0.419
Angiotensin-receptor blocker	64 (32.7%)	50 (33.1%)	0.037	0.114	30 (33.0%)	30 (30.0%)	0.005	0.422
Sacubitril-valsartan	30 (15.3%)	25 (16.6%)	0.019	0.231	14 (15.4%)	15 (16.5%)	0.008	0.342
Beta-blocker	151 (77.0%)	119 (78.8%)	0.027	0.192	71 (78.0%)	71 (78.0%)	0.005	0.417
Mineralocorticoid receptor antagonist	70 (35.7%)	59 (39.1%)	0.052	0.103	33 (36.3%)	35 (38.5%)	0.020	0.216
Digitalis	18 (9.2%)	13 (8.6%)	0.029	0.247	8 (8.8%)	8 (8.5%)	0.006	0.419
History of smoking	99 (50.5%)	81 (53.6%)	0.039	0.149	46 (50.5%)	48 (52.7%)	0.029	0.284
History of alcohol use disorder	65 (33.2%)	54 (35.8%)	0.048	0.122	31 (34.1%)	32 (35.2%)	0.021	0.237
Hypertension	140 (73.4%)	114 (75.5%)	0.037	0.198	67 (73.6%)	68 (74.7%)	0.029	0.272
Dyslipidaemia	138 (70.4%)	110 (72.8%)	0.049	0.181	65 (71.4%)	66 (72.2%)	0.018	0.202
Chronic kidney disease	38 (19.4%)	22 (14.6%)	0.089	0.086	17 (18.7%)	15 (16.5%)	0.017	0.198
Cerebrovascular disease	10 (5.1%)	9 (5.9%)	0.027	0.213	5 (5.5%)	5 (5.5%)	0.009	0.412
Chronic obstructive pulmonary disease	79 (40.5%)	65 (43.0%)	0.029	0.229	37 (40.7%)	39 (42.9%)	0.026	0.221
Atrial fibrillation	60 (30.6%)	53 (35.1%)	0.067	0.100	29 (31.9%)	31 (34.1%)	0.021	0.241
Laboratory findings at admission								
Glucose (mg/dL)	143.2 ± 16.5	150.1 ± 18.4	0.027	0.184	147.2 ± 17.2	148.9 ± 18.0	0.009	0.402
Glycated haemoglobin (%)	7.1 ± 0.5	7.2 ± 0.6	0.012	0.249	7.1 ± 0.5	7.2 ± 0.6	0.008	0.426
NT-proBNP (pg/mL)	3351 ± 921	3192 ± 808	0.042	0.184	3281 ± 817	3201 ± 801	0.011	0.307
Creatinine (mg/dL)	1.39 ± 0.48	1.31 ± 0.45	0.039	0.298	1.33 ± 0.46	1.35 ± 0.46	0.008	0.419
eGFR (mL/min/1.73 m2)	54.0 ± 19	58.0 ± 20	0.067	0.103	56.0 ± 19	57.20 ± 19	0.009	0.409
Uric acid (mg/dL)	6.4 ± 1.7	6.0 ± 1.6	0.027	0.219	6.2 ± 1.6	6.1 ± 1.6	0.008	0.385
Sodium (mmol/L)	136.0 ± 8.6	137.0 ± 9.2	0.030	0.249	136.0 ± 8.6	137.0 ± 8.9	0.009	0.407
Potassium (mmol/L)	4.7 ± 1.9	4.9 ± 1.9	0.038	0.219	4.8 ± 1.9	4.8 ± 1.9	0.004	0.459
Aspartate aminotransferase (IU/L)	23 ± 15	29 ± 16	0.029	0.237	25 ± 16	27 ± 16	0.016	0.302
Alanine aminotransferase (IU/L)	31 ± 19	37 ± 20	0.032	0.260	32 ± 19	34 ± 20	0.020	0.216
Gamma-glutamyltransferase (IU/L)	42 ± 21	53 ± 24	0.049	0.204	46 ± 22	50 ± 23	0.027	0.199

Values are shown as mean ± standard deviations, absolute values, and percentages. Standardized difference >10% (>0.1) is considered to represent a non-negligible difference. Values were considered to be statistically significant when *p* < 0.05. eGFR: estimated glomerular filtration rate; NT-proBNP: N-terminal pro-B-type natriuretic peptide; NYHA: New York Heart Association.

**Table 2:** Glycaemic control and insulin therapy of patients hospitalised for acute decompensated heart failure according to antihyperglycaemic regimen: pre- and post-propensity matching analysis.

	Pre-Propensity Matching Analysis				Post-Propensity Matching Analysis			
	Basal-Bolus (*n* = 196)	Empagliflozin-Basal (*n =* 151)	Standardised Difference	*p* Value	Basal-bolus (*n =* 91)	Empagliflozin-Basal (*n =* 91)	Standardised Difference	*p* Value
Glycaemic control								
Mean BG during hospitalisation (mg/dL)	149.5 ± 16.9	158.1 ± 20.2	0.131	0.041	152.1 ± 17.8	155.2 ± 19.7	0.014	0.289
Pre-breakfast mean BG (mg/dL)	145.1 ± 15.6	153.4 ± 18.0	0.088	0.087	150.0 ± 16.7	151.9 ± 18.4	0.028	0.192
Pre-lunch mean BG (mg/dL)	157.8 ± 19.4	169.2 ± 22.9	0.142	0.040	160.4 ± 19.4	164.5 ± 19.7	0.022	0.183
Pre-dinner mean BG (mg/dL)	153.9 ± 18.2	160.5 ± 19.2	0.069	0.068	156.4 ± 19.7	160.9 ± 19.8	0.019	0.201
Bedtime mean BG (mg/dL)	157.1 ± 19.1	168.0 ± 21.0	0.073	0.081	160.1 ± 19.9	164.2 ± 20.0	0.014	0.217
Patients with mean BG 100-140 mg/dL	40 (20.4%)	27 (17.9%)	0.091	0.089	17 (18.7%)	16 (17.6%)	0.022	0.169
Patients with mean BG 140-180 mg/dL	75 (38.3%)	61 (40.4%)	0.083	0.105	36 (39.6%)	36 (39.6%)	0.019	0.301
Patients with mean BG 180-250 mg/dL	18 (9.2%)	22 (14.6%)	0.148	0.039	11 (12.1%)	13 (14.3%)	0.016	0.284
Number of treatment failures	28 (14.3%)	32 (21.2%)	0.151	0.036	16 (17.6%)	18 (19.8%)	0.019	0.299
Day of treatment failure	2.6 ± 1.3	2.0 ± 1.3	0.068	0.208	2.6 ± 1.3	2.1 ± 1.3	0.067	0.164
Insulin therapy								
Total insulin dose (Units per day)	31.0 ± 5.5	20.3 ± 4.3	0.287	<0.001	29.0 ± 5.0	20.1 ± 4.1	0.291	<0.001
Total basal insulin dose (Units per day)	15.0 ± 2.7	16.8 ± 3.0	0.081	0.117	15.1 ± 2.9	16.1 ± 2.9	0.015	0.296
Total prandial rapid-acting insulin dose (Units per day)	10.0 ± 3.0	-	-	-	9.0 ± 3.0	-	-	-
Total supplemental rapid-acting insulin dose (Units per day)	5.5 ± 1.2	6.1 ± 1.9	0.079	0.223	5.9 ± 1.1	6.1 ± 1.8	0.010	0.311
Number of injections per day during hospital stay	4.0 ± 0.0	2.3 ± 0.7	0.273	<0.001	4.0 ± 0.0	2.3 ± 0.8	0.354	<0.001

Values are shown as mean ± standard deviations, absolute values, and percentages. A standardised difference of >10% (>0.1) is considered to represent a non-negligible difference. Values were considered to be statistically significant when *p* < 0.05.BG: blood glucose.

**Table 3:** Safety endpoints of patients hospitalised for acute decompensated heart failure according to antihyperglycaemic regimen: pre and post-propensity matching analysis.

	Pre-Propensity Matching Analysis				Post-Propensity Matching Analysis			
	Basal-Bolus (*n* = 196)	Empagliflozin-Basal (*n* = 151)	Standardised Difference	*p* Value	Basal-Bolus (*n* = 91)	Empagliflozin-Basal (*n* = 91)	Standardised Difference	*p* Value
Adverse events								
Total number	39 (19.9%)	23 (15.2%)	0.061	0.178	17 (18.7%)	14 (15.4%)	0.052	0.181
Cardiovascular	14 (7.1%)	7 (4.6%)	0.103	0.042	7 (7.7%)	5 (5.5%)	0.048	0.199
Respiratory	10 (5.1%)	6 (4.0%)	0.041	0.199	4 (4.4%)	4 (4.4%)	0.024	0.298
Infectious	7 (3.6%)	4 (2.6%)	0.058	0.187	3 (3.3%)	2 (2.2%)	0.059	0.179
Thromboembolic	2 (1.0%)	1 (0.7%)	0.034	0.210	1 (1.1%)	1 (1.1%)	0.021	0.290
Renal/Urinary	5 (2.6%)	5 (3.3%)	0.084	0.094	2 (2.2%)	2 (2.2%)	0.019	0.293
Other	1 (0.5%)	0	0.021	0.304	0	0	NA	NA
Adverse events of special interest	8 (4.1%)	9 (6.0%)	0.073	0.104	4 (4.4%)	5 (5.5%)	0.031	0.202
Worsening heart failure	12 (6.1%)	5 (3.3%)	0.109	0.043	6 (6.6%)	3 (3.3%)	0.093	0.067
Discontinuation	-	12 (7.9%)	NA	NA	-	6 (6.6%)	NA	NA
Hypoglycaemia								
Total number of hypoglycaemic episodes	46	20	0.201	0.002	24	12	0.288	<0.001
Patients with 1 hypoglycaemic episodes	30 (15.3%)	13 (8.6%)	0.265	<0.001	13 (14.3%)	8 (8.8%)	0.112	0.039
Patients with ≥2 hypoglycaemic episodes	25 (12.8%)	7 (4.6%)	0.302	<0.001	10 (11.0%)	5 (5.5%)	0.147	0.012
Hypoglycaemias incidence rate (per 100 patient-years)	17.9	6.6		<0.001	16.0	8.4		0.002
Patients with any level 1 hypoglycaemia	24 (12.2%)	10 (6.6%)	0.149	0.014	10 (11.0%)	6 (6.6%)	0.152	0.021
Patients with any level 2 hypoglycaemia	8 (4.1%)	3 (1.2%)	0.104	0.043	3 (3.3%)	2 (2.2%)	0.083	0.109
Patients with any level 3 hypoglycaemia	4 (2.0%)	1 (0.4%)	0.102	0.044	1 (1.1%)	1 (1.1%)	0.051	0.179
Length of hospital stay (days)	8.0 ± 2.5	7.5 ± 2.3	0.032	0.221	8.0 ± 2.5	7.9 ± 2.3	0.017	0.284
In-hospital death	9 (4.6%)	6 (4.0%)	0.065	0.179	4 (4.4%)	4 (4.4%)	0.014	0.301

Values are shown as mean ± standard deviations, absolute values, and percentages. A standardised difference of >10% (>0.1) is considered to represent a non-negligible difference. Values were considered to be statistically significant when *p* < 0.05. NA: not applicable.

## Data Availability

The data presented in this study are available on request from the corresponding author. The data are not publicly available due to ethical restrictions.
